# Can Unilateral Strength Training Optimize Change of Direction Mechanics and Mitigate Anterior Cruciate Ligament Injury Risk in Female Soccer Players? A Preliminary Pre–Post Intervention Study

**DOI:** 10.3390/sports13050135

**Published:** 2025-04-29

**Authors:** Alice Rogalski, Ayrton Moiroux-Sahraoui, Maria Stergiou, Maxence Pieulhet, Maurice Douryang, Florian Forelli

**Affiliations:** 1 Racing Club de Lens, CTS La Gaillette, 62065 Avion, France; rogalski.alice@gmail.com; 2 Orthopaedic Surgery Department, Évry Paris-Saclay University, 91090 Évry-Courcouronnes, France; 3 Orthosport Rehab Center, 95330 Domont, France; ayrton.moirouxsahraoui@gmail.com; 4 Orthopaedic Surgery Department, Clinic of Domont, Ramsay Healthcare, @OrthoLab, 95330 Domont, France; 5 Department of Sports Medicine, Universidad Europa de Madrid-Real Madrid, 28005 Madrid, Spain; stergioumaria89@gmail.com; 6 Olympique Lyonnais Academy, 69282 Meyzieu, France; orthosport.kine@gmail.com; 7 Department of Physiotherapy and Physical Medicine, University of Dschang, Dschang P.O. Box 96, Cameroon; douryangmaurice@gmail.com; 8 Haute-Ecole Arc Santé, HES-SO University of Applied Sciences and Arts Western Switzerland, 2800 Delémont, Switzerland; 9 Société Française des Masseurs—Kinésithérapeutes du Sport Lab, 93380 Pierrefite sur Seine, France

**Keywords:** anterior cruciate ligament injury prevention, neuromuscular adaptation, unilateral loading, change of direction, biomechanics

## Abstract

Background: Anterior cruciate ligament (ACL) injuries are a major concern in female soccer players, with change of direction (COD) movements being a key contributor to non-contact injuries. Traditional injury prevention programs have shown limited effectiveness in addressing individual biomechanical deficits. This study aimed to evaluate the effects of a 10-week unilateral strength training program on COD mechanics and ACL injury risk factors. Methods: Eleven female soccer players participated in a pre–post intervention study. Movement mechanics was assessed using the Cutting Movement Assessment Score (CMAS) at 45°, 75°, and 90° angles. The training program included unilateral lower limb strengthening exercises designed to enhance neuromuscular control and reduce biomechanical risk factors. Risk profiles were analyzed before and after the intervention. Statistical analyses included paired t-tests and effect size calculations. Results: Significant improvements in CMAS scores were observed at all COD angles (*p* < 0.05), with the largest effect at 90° (Cohen’s d = 2.1). The percentage of high-risk players decreased from 82% to 0%, while the proportion of low-risk players increased from 36% to 73%. Improved knee alignment, foot placement, and trunk stability were key contributors to the observed movement enhancements. Conclusions: Unilateral strength training may effectively improve COD mechanics and reduces biomechanical risk factors associated with ACL injuries in female soccer players. Our findings suggest that individualized training interventions can influence current injury prevention strategies.

## 1. Introduction

Female soccer players face unique challenges, particularly regarding injury risks. Among these, anterior cruciate ligament (ACL) ruptures have emerged as one of the most concerning injuries due to their long recovery time, high recurrence rate, and potential career-threatening implications [1,2].

ACL ruptures are significantly more frequent in female soccer players than in their male counterparts, with studies estimating that female athletes are four to eight times more likely to suffer from this injury [3,4]. This gender disparity in injury risk has been attributed to a combination of anatomical, neuromuscular, biomechanical, and hormonal factors [5]. Unlike male athletes, female players tend to exhibit greater knee valgus angles, lower hamstring activation relative to quadriceps, and increased ligament laxity due to hormonal fluctuations, particularly during the menstrual cycle [6,7]. These factors make female soccer players more susceptible to non-contact ACL injuries, which account for nearly 80% of all ACL ruptures [8].

Biomechanical research has identified change of direction (COD) movements as the most common mechanism leading to non-contact ACL injuries in female soccer players [6,9]. COD actions, which occur hundreds of times per match, place immense stress on the knee joint, especially when performed with poor neuromuscular control, inadequate trunk stability, or excessive ground reaction forces [10]. The peak knee abduction moment—a key biomechanical marker of ACL stress—has been found to be significantly higher in female athletes, particularly when executing side-step cutting movements at angles between 45° and 90° [6]. Furthermore, studies have demonstrated that faster approach speeds, improper foot placement, and excessive lateral trunk motion amplify ACL loading during COD movements [11,12].

Given that COD mechanics are modifiable risk factors, there is growing interest in developing training interventions that can optimize movement patterns and reduce ACL injury risk. Traditional injury prevention programs, such as FIFA 11+ and the PEP Program, have focused on neuromuscular training, proprioception, and plyometrics [13,14]. While these programs have shown some effectiveness in reducing ACL injuries, their impact remains limited, with many players struggling to adopt long-term adherence [15].

One of the primary limitations of current ACL prevention programs is their emphasis on bilateral strength training, which may not fully replicate the unilateral nature of most soccer movements [16,17]. Studies suggest that unilateral strength training could be more beneficial in enhancing neuromuscular control, improving force absorption, and stabilizing the knee joint during COD tasks [18,19]. Unilateral exercises, such as single-leg squats, step-ups, and single-leg Romanian deadlifts, have been shown to promote greater muscle activation, enhance dynamic balance, and reduce asymmetries between limbs, all of which are crucial for injury prevention and movement mechanics enhancement [20,21].

Additionally, trunk and core stability are known to play a supporting role in ACL injury prevention, particularly by enhancing postural control and reducing lateral trunk motion during high-stress movements like COD tasks [22]. Core engagement was indirectly addressed through compound unilateral exercises, such as planks, TRX rows, and landmine rotations, which promote trunk stabilization in dynamic contexts. While core training was not the primary focus of the program, its integration into functional movements may have contributed to the improvements observed in movement mechanics [23].

Despite the biomechanical relevance of unilateral strength training, few studies have directly evaluated its impact on COD mechanics and ACL injury risk in female soccer players [18,19,21]. The primary objective of this study was to evaluate whether integrating unilateral strength exercises into the conventional training regimen of female soccer players could optimize change of direction movement patterns and reduce biomechanical risk factors associated with ACL injuries.

To achieve this, we conducted a 10-week intervention study involving female soccer players, assessing their COD movement quality before and after exposure to unilateral strength training. The findings from this study aim to provide evidence-based recommendations for soccer-specific injury prevention strategies, with the ultimate goal of reducing the incidence of non-contact ACL injuries in female players.

## 2. Materials and Methods

### 2.1. Study Design

This study employed a pre–post experimental design to evaluate the effects of a 10-week unilateral strength training program on COD mechanics in female soccer players. The dependent variables included CMAS scores at 45°, 75°, and 90°, as well as the distribution of risk profile classifications (high, moderate, low) based on movement patterns.

The study was conducted in a controlled training environment, with all testing and training sessions supervised by certified strength and conditioning specialists. Ethical approval was obtained in accordance with institutional guidelines for human research ethics.

### 2.2. Participants

A total of 11 female soccer players from a second division (D2) French national league voluntarily participated in the study. While the sample size was small, previous studies have shown that significant changes in COD mechanics can be detected in cohorts of similar sizes [24]. Based on the participant classification framework by McKay et al., the players in this study can be categorized as ‘trained/sub-elite’ athletes, given their consistent training history, competitive level (D2), and weekly training frequency [25]. The intervention took place during the pre-season period, approximately eight weeks before the start of official competition, allowing for controlled integration of the strength training program alongside technical and tactical preparation.

The participants were aged between 18 and 25 years, with a minimum of 3 years of competitive soccer experience. Inclusion criteria required regular participation in team training sessions at least four times per week, absence of any neuromuscular disorder affecting movement, and the ability to perform high-intensity running and strength exercises without limitations. Exclusion criteria included any previous ACL reconstruction, ongoing musculoskeletal injuries, significant lower-limb deformities affecting movement mechanics, or missing more than two training sessions during the intervention period. All players provided written informed consent, and the study was conducted in accordance with the Declaration of Helsinki.

### 2.3. Sample Size Calculation

The sample size calculation was performed using GPower (version 3.1.9.7) for a paired t-test design. Assuming a moderate effect size (Cohen’s d = 0.5), a significance level of 5% (α = 0.05), and a statistical power of 80% (1 − β = 0.80), the analysis estimated that a minimum of 34 participants would be required to detect a significant pre–post intervention difference.

### 2.4. Strength Training Protocol

A structured 10-week unilateral strength training program was implemented to target neuromuscular control, inter-limb asymmetries, and trunk stability. Training sessions were conducted twice per week (approx. 45 min each) under the supervision of a certified strength and conditioning coach. Each session included a warm-up phase followed by four to five exercises focusing on unilateral lower-limb strength, core stability, and balance.

The program was designed with progressive overload and variation across weeks, targeting different neuromuscular objectives such as posterior chain activation, eccentric control, and dynamic trunk stability. Exercises included single-leg press, step-ups, Bulgarian split squats, TRX rows, Copenhagen planks, and landmine rotations. Detailed weekly breakdowns of exercises, dosages (sets, reps), tempos, rest intervals, and primary focuses are provided in Table 1.

The tempo was carefully prescribed to control movement execution, with eccentric emphasis (e.g., 30X0 or 40X0) to enhance braking force and neuromuscular adaptation. In the final week, training was light and recovery-oriented in preparation for post-intervention CMAS testing.

No additional training modalities were introduced during the intervention period beyond the team’s regular technical–tactical sessions, and participants were instructed to maintain consistent training and recovery habits [26].

To individualize the program, each player first completed a baseline CMAS evaluation and a unilateral strength screening (five-rep max single-leg press and Rate Perceived Exertion-based core tests). The certified strength coach then tailored the exercise selection, load, and tempo for each athlete to address their specific deficits. Female soccer players exhibiting pronounced dynamic valgus received additional sets of hip external rotator and abductor exercises, while those with poorer trunk control were prescribed extra planks and anti-rotational drills. Progression for each exercise was adjusted individually based on weekly Rate Perceived Exertion feedback and improvements in test results.

### 2.5. Testing Protocols

COD mechanics were assessed using the Cutting Movement Assessment Score (CMAS), a validated qualitative tool for evaluating movement patterns related to ACL injury risk [27]. Testing was performed at three different angles: 45°, 75°, and 90°. Each participant performed 3 trials per direction (left and right) for each COD angle, totaling 18 recorded trials per player.

**Figure 1:**
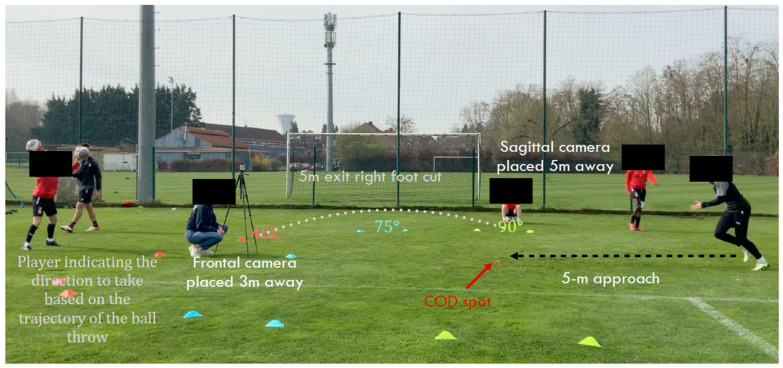
Setup and execution of the Cutting Movement Assessment Score (CMAS) Test.

Video-based motion capture was performed using Logitech high-speed camera system (Logitech, Switzerland), positioned at frontal, sagittal, and oblique angles to capture key biomechanical parameters (Figure 1). The CMAS evaluation focused on nine key biomechanical factors, including braking strategy, foot placement (defined as the mediolateral position of the foot at initial contact relative to the athlete’s center of mass. Optimal placement: contact directly under the center of mass, minimizing lever arm and frontal-plane shear forces; Suboptimal placement: landing too far medially or laterally, which increases external knee abduction moments and ACL loading), hip rotation, knee valgus, trunk position, foot orientation (defined as the rotational angle of the foot at contact (toe-in or toe-out) relative to the direction of travel. Excessive internal or external rotation (>10°) creates torsional knee loads and elevates ACL strain), final knee flexion, and weight shift mechanics (Table 2). During the CMAS assessment, excessive foot pronation (defined as medial collapse of the foot involving eversion and dorsiflexion) was qualitatively evaluated as part of the foot orientation criterion. This was based on its influence on tibial rotation and knee valgus mechanics. Although pronation is not a standalone scoring item, its presence contributed to the scoring of suboptimal foot orientation and was documented accordingly. Each of the nine biomechanical criteria was scored on a 3-point ordinal scale: 0 (optimal execution); 1 (minor deviation); and 2 (clear and repeated deficit). The total CMAS score was calculated by summing the points across all categories for each trial, with a maximum score of 18. Based on established risk classification criteria, movement profiles were categorized as follows:

- Low risk: CMAS score ≤ 4;
- Moderate risk: CMAS score = 5–6;
- High risk: CMAS score ≥ 7.

These thresholds were used to assign individual risk levels for COD angles, allowing for pre–post comparisons of injury-related movement quality.

### 2.6. Data Analysis

All data were analyzed using SPSS v27.0. Normality was tested using the Shapiro–Wilk test, and paired t-tests were used to compare pre- and post-intervention CMAS scores and strength metrics. A pre–post Student’s t-test was applied to assess differences before and after the intervention. High-risk movement profiles were analyzed pre- and post-intervention to determine reductions in the number of high-risk players. Changes in risk profile classifications (e.g., high to moderate or low risk) were assessed using McNemar’s test for paired nominal data. Effect sizes were calculated using Cohen’s d, with thresholds of 0.2 for small, 0.5 for moderate, and 0.8 for large effects [28]. A 95% confidence interval was used for all comparisons, and statistical significance was set at *p* < 0.05.

## 3. Results

### 3.1. Demographic Characteristics

The participants had a mean age of 20.3 ± 1.8 years, an average height of 167.2 ± 4.6 cm, and a mean body weight of 60.5 ± 3.9 kg.

### 3.2. Change of Direction Mechanics Improvement

After completing the 10-week unilateral strength training program, significant improvements were observed in CMAS scores across all angles. The reduction in scores was 0.73 points at 45° (*p* = 0.023, d = 0.6), 1.32 points at 75° (*p* = 0.0023, d = 1.2), and 2.23 points at 90° (*p* = 0.0001, d = 2.1). The greatest improvements were seen in 90° COD tasks, which also had the largest effect size, indicating a substantial enhancement in movement mechanics and a reduced ACL injury risk (Table 3).

### 3.3. Reduction in High-Risk Movement Profiles

Prior to training, 82% of players exhibited high-risk movement patterns in 90° COD tasks, while 55% displayed moderate-risk patterns at 75°, and 64% had moderate-risk movement profiles at 45°. Following the training intervention, none of the players remained in the high-risk category for 90° COD tasks, with 55% classified as low-risk and 45% as moderate-risk (*p* = 0.0039). A similar trend was observed at 75°, where most players shifted to a low-risk classification (*p* = 0.0313), while at 45°, 73% of players were categorized as low-risk (*p* = 0.0313) (Table 4).

## 4. Discussion

The findings of this study demonstrate a significant improvement in movement quality and reductions in biomechanical risk factors following the intervention of a 10-week training program incorporating unilateral strength exercises. This improvement is evidenced by notable increases in CMAS at 45°, 75°, and 90° angles.

### 4.1. Enhancement of Movement Mechanicses

The observed improvement in CMAS scores suggests an optimization of movement mechanics during COD tasks, likely associated with a reduction in biomechanical risk factors such as knee valgus, improper foot orientation, and inadequate trunk control [29,30,31]. These factors have previously been linked to an increased risk of ACL rupture in female athletes [10].

One of the critical risk factors for ACL injury is dynamic knee valgus, a condition where the knee collapses inward during COD tasks, increasing strain on the ACL [32]. Our study found a significant decrease in knee valgus post intervention, particularly at the 90° COD angle, where the effect size was the greatest (Cohen’s d = 2.1). This supports previous findings that emphasize the importance of hip abductor and external rotator strength in maintaining proper knee alignment [33]. Our study observed a significant reduction in knee valgus following the intervention, with the greatest improvement occurring at the 90° COD angle (Cohen’s d = 2.1). This finding aligns with prior research suggesting that strengthening the hip abductors and external rotators plays a crucial role in maintaining proper knee alignment during COD movements [33]. Unilateral exercises such as single-leg Romanian deadlifts, Bulgarian split squats, and Copenhagen planks specifically target the hip abductors and external rotators by requiring the stance limb to stabilize the pelvis and resist adduction and internal rotation forces. This focused neuromuscular challenge enhances the recruitment and strength of these muscle groups, which in turn improves frontal-plane knee alignment and reduces dynamic valgus during COD tasks. When performing a single-leg squat or deadlift, the gluteus medius and minimus must work eccentrically to prevent pelvic drop—this overloads and strengthens the hip abductors more effectively than bilateral lifts.

Hewett et al. demonstrated that neuromuscular training programs incorporating strength and plyometric exercises significantly reduce knee valgus during COD tasks [34]. Similarly, our results confirm that unilateral strength training can contribute to this improvement by specifically targeting hip stabilizers and improving neuromuscular control. This may explain why our study observed the largest reductions in CMAS scores at 90°, where knee valgus is often most pronounced due to the higher braking forces required during sharp directional changes [35].

Correct foot placement and orientation are crucial for efficient COD execution, as improper positioning can lead to excessive joint torques and increase ACL stress [36]. Beyond knee mechanics, correct foot placement and orientation are also critical for efficient COD execution, as improper positioning can lead to excessive joint torques and increased ACL stress [37]. Our study observed notable improvements in foot mechanics, with players demonstrating better foot alignment and lower excessive pronation post intervention. In our CMAS assessment, we treated pronation as an ankle/subtalar deficit rather than a foot-placement metric. Pronation (characterized by eversion plus dorsiflexion) increases tibial internal rotation and contributes to medial knee collapse, thereby elevating ACL strain. Pre-intervention, frequent excessive pronation errors were noted; post intervention, these errors decreased markedly, suggesting improved ankle–hip neuromuscular control. This reduction in pronation likely aided in lowering knee valgus and overall injury risk. Although our program did not include isolated proprioceptive drills, many of the unilateral strength exercises—such as single-leg step-ups, single-leg presses, and Bulgarian split squats—demand precise foot placement under load. Maintaining stability during these movements requires activation of the intrinsic foot muscles, tibialis posterior, and peroneal to control subtalar motion. Over the 10-week intervention, the resulting increase in strength and proprioceptive feedback within the foot–ankle complex likely improved participants’ ability to land with the foot directly under the centre of mass, reducing frontal-plane deviations and misplaced foot contacts. This mechanism explains the observed enhancements in foot placement and orientation following our strength-focused protocol.

These findings are supported by Blackburn and Padua, who reported that athletes with poor foot mechanics exhibit greater knee joint torques, predisposing them to ACL injuries [38]. By contrast, athletes who underwent neuromuscular training, including targeted foot positioning drills, showed enhanced movement mechanics and reduced ACL loading [18,39]. Our study aligns with these findings, as post-intervention assessments revealed improved foot positioning across all COD angles, contributing to enhanced overall movement quality.

Trunk stability plays a pivotal role in force absorption and distribution during COD movements. Poor trunk control has been associated with higher ACL injury rates, as excessive lateral lean and lack of core activation can increase knee joint stress [23].

Our results indicate that players demonstrated improved trunk control after the training program, aligning with previous studies emphasizing the role of core strength and neuromuscular training in ACL injury prevention [24]. Unilateral exercises, such as single-leg Romanian deadlifts, Bulgarian split squats, and Copenhagen planks, place high demands on the lateral and anti-rotational core muscles (obliques, transverse abdominis, multifidus). During these movements, the stance-leg hip and trunk must resist lateral tilt and rotation under load, leading to greater activation and strength gains in the deep spinal and abdominal stabilizers. Over the 10-week intervention, this targeted overload translated into measurable improvements in trunk stability during high-stress COD tasks. This improvement is likely due to the inclusion of unilateral strength exercises, which engage core stabilizers and enhance postural control during dynamic movements [40]. Similar findings were reported by Myer et al., who observed that athletes with stronger core engagement exhibit reduced trunk deviations and improved knee stability during COD tasks [33].

### 4.2. Impact of Unilateral Strength Training

The integration of unilateral strength training appears to have played a key role in improving COD execution and reducing ACL injury risk in this study. Unilateral exercises specifically target lower limb asymmetries, a crucial factor in injury prevention [24]. Research has shown that imbalances between dominant and non-dominant limbs can contribute to altered biomechanics, increasing ACL stress [41].

Enhanced CMAS in single-leg cutting tasks post intervention suggests improved neuromuscular control resulting from the unilateral strength training. These results align with those of Myer et al., who demonstrated that single-leg strength training leads to more symmetrical movement patterns, reducing excessive knee valgus and hip adduction during COD tasks [33].

Additionally, our findings support previous research emphasizing that unilateral strength training improves force application during deceleration and propulsion phases, leading to smoother and safer movement transitions [4]. Improvements in CMAS scores indicate enhanced single-leg cutting mechanics and neuromuscular control as a result of the unilateral strength training. This suggests that unilateral training not only enhances strength but also refines biomechanical control, which is crucial for reducing ACL injury mechanisms [35].

The effectiveness of unilateral strength training in our study further supports the argument that traditional injury prevention programs may be insufficient without a focus on individual limb strength. While programs such as the FIFA 11+ emphasize general neuromuscular training, they often do not address unilateral deficits, which are critical contributors to ACL injuries in female athletes [15]. Our findings suggest that incorporating individualized unilateral strength exercises may lead to greater improvements in movement mechanics and injury prevention compared to broad-based, non-individualized programs.

### 4.3. Comparison with Existing Injury Prevention Programs

Widely used ACL injury prevention programs, such as FIFA 11+, have demonstrated variable effectiveness in female athletes [42]. While these programs incorporate essential neuromuscular training components, they often lack an emphasis on individualized strength training, particularly in addressing unilateral imbalances [15].

Research has shown that female athletes often exhibit neuromuscular deficits, such as delayed muscle activation and weaker hip abductors, contributing to inefficient COD mechanics [33]. Our study supports these findings, as players demonstrated improved movement control and lower-risk biomechanics post intervention. Our findings demonstrate that including unilateral strength training is an effective component of ACL injury prevention strategies, although direct comparisons with other programs are beyond the scope of this study.

### 4.4. Consideration of Risk Profiles

The analysis of risk profiles before and after the intervention revealed a significant reduction in the percentage of high-risk players, dropping from 82% to 0%, while the percentage of low-risk players increased from 36% to 73%. These results suggest that the training program effectively modified biomechanical risk factors associated with ACL injuries.

Previous studies have similarly demonstrated that targeted neuromuscular interventions can significantly alter injury risk profiles in female athletes [34,43]. Myer et al. found that athletes with higher initial movement deficiencies benefited the most from structured strength and stability training, which aligns with our findings showing that those classified as high-risk before the intervention experienced the greatest improvements [33].

Research has also highlighted that changes in risk profiles correlate with reductions in actual ACL injury rates over time [24]. This suggests that the shifts observed in our study, particularly the decrease in high-risk classifications, may translate to a lower likelihood of ACL injuries in competition settings. Furthermore, Kristianslund et al. reported that improvements in neuromuscular control, especially at high-stress movement angles, such as 90° COD, are critical for long-term injury prevention, reinforcing the relevance of our results [35].

The findings also emphasize the importance of individualizing training programs based on initial movement screenings. While many ACL prevention programs take a generalized approach, our study supports the perspective that assessing individual risk profiles and tailoring interventions accordingly may lead to better outcomes, as also suggested by Padua et al. [36].

## 5. Limitations

Despite the promising findings of this study, several limitations must be acknowledged. First, the sample size was relatively small (n = 11), which may limit the generalizability of the results to a broader population of female soccer players. Future studies should aim to include larger sample sizes to enhance statistical power and external validity.

Second, the absence of a control group prevents a direct comparison between the intervention and standard training protocols. While the significant improvements observed suggest that unilateral strength training played a crucial role in enhancing movement mechanics, a control group would provide stronger evidence regarding the causal relationship between the intervention and performance outcomes.

Third, the study primarily focused on short-term improvements in CMAS scores and biomechanical risk factors. Although these metrics are important indicators of ACL injury risk, long-term follow-up is necessary to determine whether the observed improvements translate into reduced ACL injury rates in real-game scenarios. Future research should incorporate longitudinal designs to assess injury incidence over multiple seasons.

Additionally, the study relied on video-based movement assessments for CMAS scoring, which, while effective, may lack the precision of three-dimensional motion capture systems. More advanced biomechanical analysis tools could provide deeper insights into movement kinematics and further validate the observed improvements.

Lastly, the training program was designed to integrate unilateral strength exercises; however, variations in individual training adherence and external factors, such as fatigue and workload management, were not controlled. Future research should explore the interplay between strength training, fatigue, and neuromuscular control to optimize ACL injury prevention strategies.

## 6. Conclusions and Practical Implications

The results of this study hold valuable implications for coaches, sports scientists, and athletic trainers aiming to enhance COD mechanics and reduce injury risk in female soccer players. The inclusion of unilateral strength exercises in training regimens appears to be a promising approach for improving movement mechanics and reducing biomechanical risk factors associated with ACL injuries.

Our data indicate that individualized neuromuscular assessments may help to identify players at higher risk of ACL injuries. By incorporating unilateral strength training into structured training programs, practitioners can specifically target movement asymmetries and enhance single-leg stability, which are critical for effective COD execution. Given that female athletes are predisposed to ACL injuries due to anatomical and neuromuscular factors, training strategies that address these deficits can provide significant injury prevention benefits.

Furthermore, teams should consider integrating unilateral strength training as a core component of their injury prevention protocols, supplementing existing prevention program such as FIFA 11+. The findings from this study suggest that current general injury prevention programs may not adequately address individual biomechanical risk factors, whereas tailored interventions incorporating unilateral exercises can yield more substantial improvements in movement quality and overall athletic performance.

## Figures and Tables

**Table 1:** Weekly breakdown of exercises, dosages, and objectives.

Week	Main Exercises (Examples)	Sets	Reps	Tempo	Rest	Primary Focus
Week 1	Single-leg press, Squat, Side plank, TRX face pull	4	10	30X0	30 s	Core stability, general activation
Week 2	Single-leg deadlift, Step-up, Unilateral TRX row, Star side plank	3–4	8–10	30X0	30–45 s	Posterior chain, balance
Week 3	Landmine rotation, Copenhagen, Squat, Plank with row	4	10–12	3010	30 s	Dynamic core, adductor strength
Week 4	Eccentric leg press, Bulgarian split squat, TRX face pull, Front plank + TRX	3–5	8–12	40X0	30–45 s	Braking force, eccentric control
Week 5	Single-leg deadlift, Star side plank, TRX row, Landmine rotation	4	10	30X0	30 s	Hip and trunk stability
Week 6	Hip thrust, Single-leg press, Bench press, Lateral TRX pull	3	10–12	3010	30–60 s	Global strength, stability
Week 7	Alternating step-ups, Lateral raises, Copenhagen, TRX	4	10	30X0	30 s	Inter-limb symmetry
Week 8	Heavy single-leg deadlift, Slow-tempo squat, Instability TRX, Dynamic plank	3–5	8–10	3010/40X0	30–45 s	Motor control, maximal force
Week 9	Functional circuit (step-up, TRX, press, rotation plank)	3 rounds	30 s/exercise	N/A	1 min/round	Coordination, technical transfer
Week 10	Mobility, light activation, static core work	2–3	12–15	Smooth	30 s	Recovery before CMAS re-test

Abbreviations—Dosage and Tempo Explained: Sets: Number of complete sets performed. Reps: Number of repetitions per set. Tempo: Timing for movement phases (detailed below). Rest: Recovery time between sets or rounds. Tempo Codes: 30X0—3 s eccentric, no pause, explosive concentric, no pause. 3010—3 s eccentric, no pause, 1 s concentric, no pause. 40X0—4 s eccentric, no pause, explosive concentric, no pause. N/A—Not applicable (time-based efforts). Smooth—Light, continuous, recovery-focused movement.

**Table 2:** Key biomechanical factors in CMAS assessment.

Biomechanical Factor	Description
Braking Strategy	Trunk inclination, center of mass control during deceleration
Foot Placement	Position of foot relative to body and center of mass
Hip Rotation	Internal rotation at initial contact and during push-off
Knee Valgus	Medial displacement of the knee at initial ground contact
Trunk Position	Lateral lean deviations that affect balance and force distribution
Foot Orientation	Angle of the foot relative to the target direction. Includes assessment of medial foot collapse and excessive pronation as contributors to rotational alignment deficits.
Final Knee Flexion	Degree of knee flexion at push-off phase
Weight Shift Mechanics	Distribution of body weight during deceleration and push-off
Ground Contact Time	Duration of foot contact with the ground during COD execution

**Table 3:** CMAS scores before and after training.

Change of Direction Angle (°)	Pre-Test Mean Score	Post-Test Mean Score	*p*-Value	Cohen’s d 95% CI
45°	3.55 ± 1.2	2.82 ± 1.0	0.023	0.6
75°	4.55 ± 1.4	3.23 ± 1.1	0.0023	1.2
90°	5.68 ± 1.6	3.45 ± 1.3	0.0001	2.1

Note: CI; Confidence Interval.

**Table 4:** Distribution of risk profiles before and after training.

COD Angle	Pre-Intervention High	Pre-Intervention Moderate	Pre-Intervention Low	Post-Intervention High	Post-Intervention Moderate	Post-Intervention Low	*p*-Value
**90°**	82%	18%	0%	0%	45%	55%	0.0039
**75°**	45%	55%	0%	9%	27%	64%	0.0313
**45°**	0%	64%	36%	0%	27%	73%	0.0313

## Data Availability

The data that support the findings of this study are available on reasonable request from the corresponding author. The data are not publicly available due to confidentiality agreements with partner institutions and the presence of sensitive personal or medical information related to athletes.
